# Supplementary material for: A Systematic Review of the Distribution of Tick-Borne Pathogens in Wild Animals and Their Ticks in the Mediterranean Rim between 2000 and 2021
Source: Microorganisms. 2022 Sep 16;10(9):1858. doi: 10.3390/microorganisms10091858 (PMC9504443; doi:10.3390/microorganisms10091858)
Supplement: Supplementary file 1 [file microorganisms-10-01858-s001.zip › microorganisms-1866203-supplementary.pdf]

Table S1. TBP-positive engorged ticks collected from wild animals and their distribution in the Mediterranean Rim

**Tick genus**	**Number of tick species and number of TBPs detected per species**		**Stage**	**Number of countries with data and countries**		**Number of tick host species with data, and host species**		**Number of pathogenic genera/species and pathogens detected in ticks (^{b}bacteria, ^{p}parasite, ^{v}virus)**	
Ixodes	8	*I. acuminatus* (5)<br>*I. festai* (2)<br>*I. frontalis* (4)<br>*I. hexagonus* (2)<br>*I. ricinus* (32)<br>*I. simplex* (1)<br>*I. ventalloi* (5)<br>*I. vespertilionis* (3)	Adults, nymphs, and larvae	7	Cyprus, France, Greece, Italy, Slovenia, Spain, and Turkey	23	Badgers, bats, birds, chamois, fallow deer, genets, hares, hedgehogs, jackals, lizards, lynxes, martens, mongooses, mouflons, polecats, red deer, red foxes, rodents, roe deer, weasels, wild boars, and wolves	39	*Anaplasma* spp.^{b}, *A. marginale*^{b}, *A. phagocytophilum*^{b}, *A. ovis*^{b}, *Bartonella* spp.^{b}, *B. acomodis*^{b}, *B. bacilliformis*^{b}, *B. bovis*^{b}, *B. chomelli*^{b}, *B. tamiae*^{b}, *B. tribocorum*^{b}, *B. vinsonnii berkoffi*^{b}, *Babesia* spp.^{p}, *Ba. capreoli*^{p}, *Ba. microti*^{p}, *Ba. ovis*^{p}, *Ba. rodaini*^{p}, *Ba. venatorum*^{p}, *Bo. afzeli*^{b}, *Bo. burgdoferi* s.l.^{b}, *Bo. burgdoferi* s.s.^{b}, *Bo. garinii*^{b}, *Bo. lusitaniae*^{b}, *Bo. spielmanii*^{b}, *Bo. valaisiana*^{b}, *Bo. turdi*^{b}, CCHF^{v}, *C. burnetii*^{b}, *Candidatus* Noehrlichia mikurensis^{b}, *Rickettsia* spp.^{b}, *R. aeschlimannii*^{b}, *R. africae*^{b}, *R. helvetica*^{b}, *R. hoogstraalii*^{b}, *R. monacensis*^{b}, *R. sibirica*^{b}, *R. slovaca*^{b}, *Theileria* spp^{p}., and *T. annae*^{p}
Rhipicephalus	6	*Rh (B). annulatus* (1)<br>*Rh. bursa* (14)<br>*Rh (B). kohlsi* (1)<br>*Rh. ovis* (1)<br>*Rh. pusillus* (2)<br>*Rh. sanguineus* s.l. (18)	Adults, nymphs, and larvae	7	Cyprus, Greece, Israel, Italy, Palestine, Spain, and Turkey	15	Birds, fallow deer, genets, hares, hedgehogs, jackals, lynxes, martens, mongooses, mouflons, red deer, red foxes, roe deer, wild boars, and wolves	22	*Anaplasma* spp.^{b}, *A. marginale*^{b}, *A. phagocytophilum*^{b}, *A. ovis*^{b}, *Bartonella* spp.^{b}, *Babesia* spp.^{p}, *Ba. rodaini*^{p}, *Bo. burgdoferi* s.l.^{b}, *Ch. abortus*^{b}, *C. burnetii*^{b}, *Ehrlichia* spp.^{b}, *E. canis*^{b}, *Rickettsia* spp.^{b}, *R. massiliae*^{b}, *R. slovaca*^{b}, *Candidatus* R. barbariae^{b}, *R. sibirica mongolitimonae*^{b}, *Candidatus* R. goldwasserii^{b}, *Theileria* spp.^{p}, *T. buffeli*^{p}, *T. orientalis*^{p}, *T. ovis*^{p}, and *T. sergenti*^{p}
Haemaphysalis	5	*H. adleri* (1)<br>*H. erinacei* (1)<br>*H. parva* (3)<br>*H. punctata* (11)<br>*H. sulcata* (5)	Adults, nymphs, and larvae	7	Cyprus, Greece, Israel, Italy, Slovenia, Spain, and Turkey	13	Birds, fallow deer, golden jackals, hares, hedgehogs, lizards, martens, mouflons, red deer, red foxes, rodents, roe deer, and wild boars	21	*Anaplasma* spp.^{b}, *A. phagocytophilum*^{b}, *A. ovis*^{b}, *B. bacilliformis*^{b}, *B. bovis*^{b}, *B. chomelli*^{b}, *Ba. crassa*^{p}, *Ba. rossi*^{b}, *Borrelia* spp.^{b}, *Bo. garinii*^{b}, *Ch. abortus*^{b}, *C. burnetii*^{b}, *E. canis*^{b}, *Rickettsia* spp.^{b}, *R. aeschlimannii*^{b}, *R. hoogstraalii*^{b}, *R. massiliae*^{b}, *R. monacensis*^{b}, *R. slovaca*^{b}, and *Candidatus* R. goldwasserii^{b},
Hyalomma	6	*Hy. aegyptum* (4),<br>*Hy. anatolicum excavatum* (1)<br>*Hy. detritum* (1)<br>*Hy. lusitanicum* (1)<br>*Hy. marginatum* (13)<br>*Hy. rufipes* (3)	Adults, nymphs, and larvae	10	Algeria, Cyprus, Greece, France, Israel, Italy, Morocco, Palestine, Spain, and Turkey	12	Birds, fallow deer, hares, hedgehogs, lynxes, mouflons, red deer, red foxes, rodents, roe deer, tortoises, and wild boars	16	*A. marginale*^{b}, *A. phagocytophilum*^{b}, *Babesia* spp.^{p}, *Ba. occultans*^{p}, CCHF^{v}, *C. burnetii*^{b}, *Ehrlichia* spp.^{b}, *Rickettsia* spp.^{b}, *R. aeschlimannii*^{b}, *R. africae*^{b}, *R. raoultii*^{b}, Tamdy orthonairovirus^{v}, *Theileria* spp.^{p}, *T. buffeli*^{p}, *T. orientalis*^{p} and *T. sergenti*^{p}
Dermacentor	1	*D. marginatus* (12)	Adults, nymphs, and larvae	5	Algeria, France, Italy, Slovenia, and Spain	10	Chamois, hares, fallow deer, jackals, martens, red deer, red foxes, rodents, roe deer, and wild boars	12	*A. marginale*^{b}, *A. phagocytophilum*^{b}, *B. bovis*^{b}, *B. chomelii*^{b}, *Babesia* spp.^{p}, *Bo. burgdoferi* s.l.^{b}, *Ehrlichia* spp.^{b}, *E. canis*^{b}, *Rickettsia* spp.^{b}, *R. raoultii*^{b}, *R. slovaca*^{b}, and *Theileria* spp.^{p}
Amblyomma	1	*Amblyomma* sp. (1)<br>*Am. marmoreum* (2)	Adults, nymphs, and larvae	3	Greece, Israel, and Italy	1	Birds	2	*Ehrlichia* spp.^{b} and *R. aeschlimannii*^{b}
Ornithodoros	3	*O. erritacus* (3)<br>*O. maritimus* (1)<br>*O. normandi* (1)	Adults	3	Morocco, Spain, and Tunisia	2	Bats and rodents	5	*Bo. crocidurae*^{b}, *Bo. hispanica*^{b}, *Bo. merionesi*^{b}, *Candidatus* R. Africa septentrioalis^{b}, and Maeban/Maeban-like virus^{v}
Argas	2	*A. transgariepinus* (1)<br>*A. vespertilionis* (2)	Adults	1	Italy	1	Bats	2	*Bartonella* spp.^{b} and *Rickettsia* spp.^{b}
Unknown	X	X	X	3	Greece, Palestine, and Turkey	3	Birds, lizards, and rodents	3	*Rickettsia* spp.^{b}, *R. aeschlimannii*^{b}, and *R. africae*^{b}

Table S2: TBP diversity in wild animals and their ticks from the Mediterranean Rim. *Wild carnivores: Badger, Genet, Golden jackal, Marten, Mongoose, Otter and Wolf

**Animal name (Number of countries with data available)**	**Wild boar (6)**	**Red deer (5)**	**Birds (4)**	**Rodent (7)**	**Wild carnivores* (6)**	**Red fox (5)**	**Tortoise (2)**	**Fallow deer (2)**	**Roe deer (1)**	**Hedgehog (4)**	**Mouflon (2)**	**Lizard (2)**	**Chamois (2)**	**Hare (3)**	**Bat (1)**	**Porcupine (2)**	**Swamp deer (1)**
**Pathogens found in ticks**																	
**Number of studies**	26	24	21	18	13	12	12	11	8	8	7	4	3	2	2	0	0
**Number of pathogens found in ticks**	Bacteria: 9 Parasite: 5	Bacteria: 15 Parasite: 1 Virus: 1	Bacteria: 16 Parasite: 4 Virus: 2	Bacteria: 17 Parasite: 3 Virus: 1	Bacteria: 8	Bacteria: 8 Parasite: 5	Bacteria: 4 Virus: 1	Bacteria: 9 Parasite: 1	Bacteria: 10 Parasite: 4	Bacteria: 6	Bacteria:7 Parasite: 4	Bacteria: 9	Bacteria: 2	Bacteria: 5	Bacteria: 4		
**Percentage of TBPs in ticks**	27.9%	26.2%	36.1%	34.4%	13.1%	21.3%	9.9%	16.4%	22.9%	9.9%	18%	14.8%	3.3%	8.2%	6.6%	0%	0%
**Pathogens found in wild animals**																	
**Number of studies**	11	22	4	28	11	13	2	6	12	0	1	3	3	3	0	3	2
**Number of pathogens found in animals**	Bacteria: 3 Parasite:2 Virus:2	Bacteria: 2 Parasite: 4	Bacteria: 5	Bacteria: 20 Parasite: 2 Virus: 1	Bacteria:2 Parasite:5	Bacteria: 3 Parasite: 5	Parasite: 1 Virus: 1	Bacteria: 1	Bacteria: 2 Parasite: 6 Virus 2	0	Bacteria: 3	Parasite:2	Parasite: 3	Parasite: 2	0	Bacteria: 1	Bacteria: 2
**Percentage of TBPs in animals**	14%	12%	10%	46%	14%	16%	4%	2%	20%	0%	6%	4%	6%	4%	0%	2%	4%
